# Short‑term effects of pregabalin plus exercise therapy on pain, emotional status, physical function and nociceptive responses in patients with fibromyalgia

**DOI:** 10.3892/mi.2023.101

**Published:** 2023-08-09

**Authors:** Onur Velioglu, Mustafa Turgut Yildizgoren, Halil Ogut, Hayal Guler, Ayse Dicle Turhanoglu

**Affiliations:** Department of Physical Medicine and Rehabilitation, Faculty of Medicine, Mustafa Kemal University, Alahan, Hatay 31001, Turkey

**Keywords:** fibromyalgia, nociceptive flexor reflex, pregabalin, aerobic exercise, resistance training

## Abstract

The aim of the present study was to investigate the effects of pregabalin plus exercise vs. pregabalin treatment alone on the electromyographic nociceptive flexion reflex (NFR) threshold in patients with fibromyalgia (FM). For this purpose, the present study included a total of 40 patients diagnosed with FM according to the American College of Rheumatology 2010 criteria. The patients were divided into two groups as follows: Group 1 received pregabalin treatment only and group 2 received exercise therapy in addition to pregabalin treatment. Assessments were made at baseline and at the 1st month using a visual analog scale (VAS) to measure pain, the Fibromyalgia Impact Questionnaire (FIQ) to measure the severity of FM, Beck's Depression Inventory (BDI) to measure depression and the NFR to measure the compressive forces on peripheral nerves. In both groups, the NFR threshold following treatment was significantly higher than that at the baseline results (P#x003C;0.001). There was no significant difference between the groups as regards the difference from pre- to post-treatment NFR threshold values (P=0.610 and P=0.555, respectively). There was a strong, negative correlation between the pre-treatment NFR threshold and VAS resting, VAS motion and FIQ scores (Rho=-0.62, Rho=-0.69 and Rho=-0.60, respectively). There was a moderate negative correlation between the pre-treatment NFR threshold and BDI scores (Rho=-0.35). On the whole, the present study demonstrates that in the treatment of FM, pregabalin improves the clinical scores and leads to an increase in the NFR threshold. Herewith, it should be noted that short-term exercise therapy does not appear to provide additional benefits.

## Introduction

Fibromyalgia (FM) is a chronic, non-inflammatory, soft-tissue disorder, characterized clinically by widespread pain, stiffness, fatigue and non-restorative sleep, which affects 2-8% of the general population (1,2). According to the American College of Rheumatology 2010 classification criteria, there are 19 areas of the body which constitute the most painful regions. Furthermore, for a definitive diagnosis there should be some symptoms for at least 3 months, which cannot be explained by any other health disorder (3).

As the etiology and pathogenesis of FM have not yet been fully elucidated, treatment is usually symptomatic. Both pharmacological and non-pharmacological treatments are applied for symptoms, such as pain, fatigue, related depression and sleep difficulties (4,5). Several treatment approaches have been described, including drug therapy, exercise, physiotherapy, spa therapy, acupuncture, diet and cognitive-behavioral therapies (5). For patients with severe pain, pharmacological treatment with duloxetine, pregabalin, or tramadol should be considered, and for those with sleep disturbances, amitriptyline, cyclobenzaprine, or pregabalin have been suggested. Rather than individual therapies, multimodal rehabilitation has been found to improve various short-term outcomes in patients with severe disability (5). Pregabalin became the first drug Food and Drug Administration-approved drug for specifically treating FM and varying responses have been reported due to the heterogeneity of the FM population (6). Pregabalin, which is a potent inhibitory ligand for the calcium channel α2-delta subunit in the central nervous system, has analgesic and anxiolytic properties in addition to anti-convulsant activity. Patients with FM treated with pregabalin have exhibited central sensitization. Previous clinical trials on pregabalin have reported a significant reduction in pain, improvements in sleep quality and a generally improved health-related quality of life in the majority of patients with FM (6,7). Studies on other patient populations have also demonstrated that exercise programs are beneficial for patients with FM (8,9). These programs consisting of stretching exercises, strengthening and aerobic conditioning have been included as part of standard treatment protocols.

The nociceptive flexion reflex (NFR) is a polysynaptic withdrawal reflex that occurs following the stimulation of nociceptive A-delta afferents (10). The level of stimulation needed to obtain this reflex can be utilized as an objective marker of nociception in patients, and allows for the exploration of the pain processing pathways at central levels (10).

Therefore, the aim of the present study was to assess the effects of pregabalin plus exercise vs. pregabalin treatment alone on the electromyographic NFR threshold in patients with FM and to determine the associations between the NFR, and the visual analog scale (VAS), Beck's Depression Inventory (BDI) and fibromyalgia impact questionnaire (FIQ) scores.

## Materials and methods

### Study population

**Figure 1:**
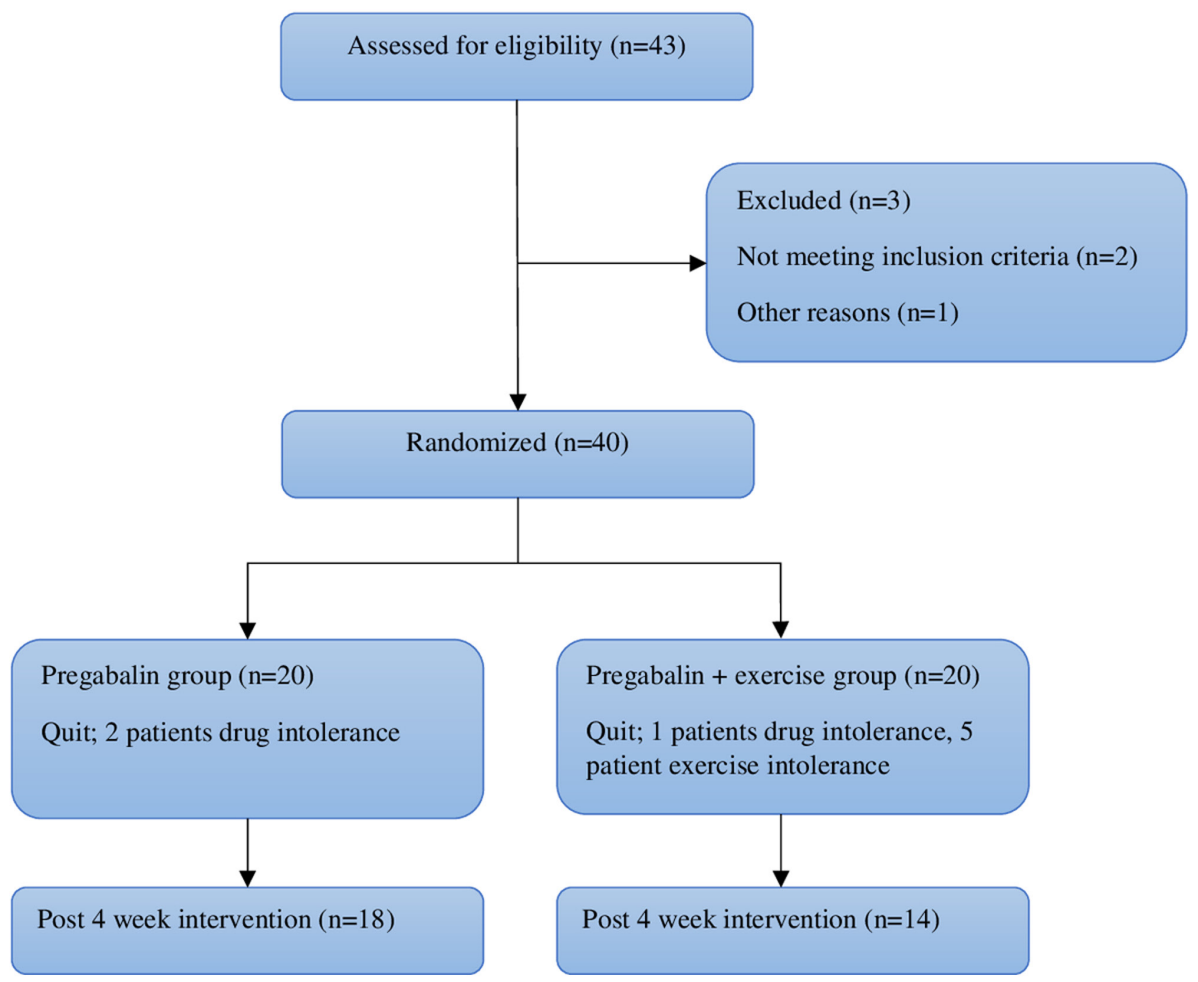
Flowchart of the present study.

The sample of the present study consisted of 40 patients who met the inclusion criteria of 43 patients between the ages of 18 and 50 with a confirmed diagnosis of FM according to the American College of Rheumatology 2010 criteria, who referred to the Physical Medicine and Rehabilitation Outpatient Clinic of Hatay Mustafa Kemal University Hospital (Alahan, Turkey) between January, 2016 and July, 2016. The patients were randomly divided into two groups with 20 patients in each (group 1, pregabalin group; group 2, pregabalin + exercise group). Randomization was performed by generating random numbers as group 1 and group 2. In groups 1,2 patients withdrew from the study due to drug intolerance and in group 2, 6 patients withdrew due to drug (1 patient) and exercise intolerance (5 patients). Eventually, the study was completed with 18 patients in group 1 and 14 patients in group 2. The study flowchart is illustrated in Fig. 1.

### Criteria for inclusion and exclusion

Patients with a history of cardiac, respiratory, neurological, psychiatric, malignancy and musculoskeletal problems including osteoarthritis, joint destruction, spinal degeneration, rheumatological diseases or trauma within the prior 3 months that may disrupt the exercise program were excluded from the study. Patients who were already being treated for FM at the time of the study initiation or within the prior 3 months, and those with smoking and alcohol use were also excluded. A clinical assessment was made on a detailed, standardized form by a physician blinded to the groups.

### Patient data

The demographic and clinical data including age, body mass index, disease duration and the number of tender points of the patients were recorded. The Ethics Committee of Hatay Mustafa Kemal University approved the study and all subjects provided written informed consent (31.03.2017/06). The study followed the Declaration of Helsinki.

### Treatment and assessment of parameters

Treatment with pregabalin for all patients in groups 1 and 2 was commenced at 75 mg twice a day in the first week then at 150 mg twice a day thereafter. Patients in group 2 also underwent a combined exercise program three times a week for 5 weeks (15 times in total) in the clinic under supervision of a physiotherapist. The program consisted of a warm-up using a treadmill for 10 min, aerobic exercises for 15 min, strengthening exercises for 15 min, and stretching and relaxation exercises for 15 min. The evaluation of the patients was made by a single researcher blinded to the groups both at baseline, and at the 1-month follow-up examination. The assessment parameters were calculated as follows: i) By applying ~4 kg/cm^2^ of thumb pressure to the predefined 18 tender points, painful points were identified and the total number was recorded for each patient; ii) pain intensity was assessed on a VAS. Using a 10-cm long line, patients recorded the level of pain on the scale where 0 represented ‘no pain’ and 10 ‘the most severe pain’; iii) BDI was used to assess the intensity of depressive symptoms. This is a 21-item self-report questionnaire often used to assess depression in patients with chronic pain, with higher total points indicating more severe depression (11); iv) the FIQ was used to score functional disability (12). The FIQ has 10 sub-scales of physical functioning, overall well-being, work missed, job difficulty, pain, fatigue, morning tiredness, stiffness, anxiety and depression. FIQ scores range from 0 to 100, with higher scores indicating a greater negative impact of FM; v) the NFR assessment was applied using a standardized, validated procedure (13). NFR assessment were performed by the same physician (ADT), who was blinded to the assignment of the groups at the beginning and at the end of the study. Repeated electrical stimuli were applied to the sural nerve endings, using an up-down staircase method, with stimulation intensity beginning at 0-mA and increasing in 4-mA increments until the detection of the first reflex. When the first NFR was detected, the stimulation intensity was decreased in 2-mA steps until the reflex disappeared. This process was repeated with stimulation intensity adjusted upward and downward in 1-mA increments until the NFR appeared and disappeared two more times. The NFR threshold was calculated as the average of the peaks and troughs of the stimulation intensities which produced the second and third occurrence of the NFR. Therefore, a higher NFR threshold value indicated that higher stimulus intensities were required to evoke a consistent reflex response. During the NFR threshold test, the patients rated the pain sensation of each electrical stimulus using a scale of 0-100, where 0 indicated no pain and 100 indicated extremely pain.

### Statistical analysis

Data analysis was conducted using SPSS version 20.0 (IBM Corp.). Data are presented as the mean ± standard deviation (SD) and median (min-max) values, and categorical data as number (n) and percentage (%). Data normality between groups was evaluated using the Shapiro-Wilk test. If the data was not normally distributed, the Mann-Whitney U test was used. In the comparisons the scores before and after treatment, the Wilcoxon signed-rank test was used according to the distribution of the data. Correlations between variables were evaluated using Spearman's correlation analysis.

The power analysis for the estimation of the sample size was unable to be performed; however, the post hoc power analysis using the G*Power^©^ 3.1 program (Heinrich-Heine-Universität Düsseldorf, Düsseldorf, Germany) was performed. The present study was performed with 32 patients with FM (group 1, n=18 vs. group 2, n=14 patients). The Wilcoxon signed-rank test was used for the comparisons if the two groups revealed an effect size of d=8.0 and the power of the study was calculated as 99% with 5% type 1 error.

## Results

Of the 20 patients in group 1, 2 patients withdrew from the study due to drug intolerance. In group 2, a total of 6 patients withdrew; 5 due to exercise intolerance and 1 due to drug intolerance. Therefore, the 5-week intervention period of the study was completed by 32 patients. The demographic and clinical characteristics of the study subjects are summarized in Tables I and II. No statistically significant differences were determined between the groups in terms of age, body mass index and duration of the disease (Table I). When the groups were compared with each other, no statistically significant differences were found between the variables at baseline, and at the 1-month evaluation (all P> 0.05) (Table II). Both groups 1 and 2 exhibited significant improvements in activity and resting VAS scores, FIQ, BDI and NFR scores after 1 month of the therapy program (all P#x003C;0.05).

The correlations between clinical measurements, pain scores, depression scores and NFR scores obtained at baseline and at 1 month after treatment are presented in Table III. At baseline, a strong negative significant correlation was found between NFR and pain (resting and activity) scores and physical function (Rho=-0.62, Rho=-0.69 and Rho=-0.60, respectively). At 1 month following treatment, in group 1, a strong negative significant correlation was identified between the NFR scores, pain (resting and activity) scores and FIQ scores (Rho=-0.82, Rho=-0.61 and Rho=-0.68, respectively). In group 2, a strong negative significant correlation was detected between the NFR scores and pain and FIQ scores (Rho=-0.66 and Rho=-0.66, respectively).

## Discussion

The present study investigated the effects of the combination of pregabalin and exercise on the NFR threshold in patients with FM. The results demonstrated a significant improvement in the NFR threshold at the 1st month after treatment. The results of the present study indicated that the treatment in both groups had positive effects on the NFR threshold of patients with FM. A significant improvement was also observed in the tender points, VAS, BDI and FIQ scores at the 1st month after treatment. There were no significant differences between pregabalin and pregabalin-exercise combination as regards efficacy.

The patients with FM have a combined symptom experience with widespread pain, sleep disturbance, fatigue and an impaired work capacity. The management consists of multidisciplinary treatment using pharmacological and non-pharmalogical strategies. Exercise therapy, including aerobic exercise, group exercise, resistance and strength training, tai chi and yoga may help prevent chronic pain and fatigue. Exercise may also help with other symptoms of FM, including depression, difficulty concentrating and sleep issues (8,9).

Aerobic exercise, such as running or brisk walking, can help with a number of symptoms of FM. Resistance training also strengthens the muscles and can improve FM symptoms. In the present study, the combination of aerobic and strengthening exercises was preferred, the effectiveness of which has been shown in previous studies (14,15). Standard strengthening or aerobic exercise is an essential non-pharmacological management method. Moreover, pain control is another critical achievement of exercise, which appears to be a secondary consequence of the improvement in muscle strength and endurance. It is known that exercise increases endorphin levels, and has antioxidant and anti-inflammatory effects. In the present study, the lack of additional benefits in symptoms with exercise may be explained by the short follow-up period.

The NFR is mediated by an anatomic substrate comprised of a complex network of interneurons that are located at various levels of the spinal cord, and these spinal interneurons are modulated by supraspinal pathways (13). Studies that have used the NFR to show the excitability of dorsal horn neurons of the spinal cord, which are formed with peripheral C fibers (a nociceptive afferent), have reported that this excitability in patients with FM causes central sensitization and chronic pain (16). Furthermore, central sensitization has been determined in patients with FM using NFR as an objective assessment of spinal nociception (16). For this reason, NFR is used in the management of central sensitization and descending pain control systems. In addition, the NFR procedure has been shown to be a valuable tool used to evaluate pharmacologically active therapeutic agents at the spinal level.

To the best of our knowledge, there are only a few studies available to date evaluating the effect of FM treatment on the NFR threshold. In a previous study comparing cognitive behavioral therapy with other medical treatments, the NFR thresholds increased significantly in the cognitive behavioral treatment group following therapy, whereas a decrease in the NFR threshold was found in the medical treatment group. Allowing both groups to use pain relief medication may have affected the NFR responses (17). In the present study, pain medication was not permitted, apart from pregabalin, as it could affect the NFR threshold. In another study, amitriptyline was shown to lead to an increase in NFR threshold (18). Matthey *et al* (19) demonstrated that milnacipran exerted a predominantly supraspinal analgesic effect, as evidenced by the significant clinical benefits and the absence of changes in the NFR threshold in patients with FM. According to the findings of the study by Matthey *et al* (19), although milnacipran led to a reduction in pain intensity and VAS scores, it did not modulate thermal allodynia and potentially restored normal diffuse noxious inhibitory control activity. The findings of the present study have demonstrated that the treatment in both groups had positive effects on the NFR threshold. The improvement in the NFR threshold following treatment with pregabalin may be interpreted as reduced central sensitization due to pregabalin. The changes in the NFR threshold values following treatment were similar in both groups; thus, it can be stated that exercise does not appear to add to the efficacy of the treatment. This may have been due to the short-term exercise therapy. Further studies with longer durations of exercise regimens are required to investigate this aspect.

Desmeules *et al* (16) found lower baseline NFR threshold values (average, 22.7 mA) in patients with FM vs. healthy controls. In another study which compared patients with FM with healthy controls, the NFR threshold value in patients with FM was found to be 12 mA on average (20). In the present study, the mean NFR threshold was determined as 11.2 mA in the patients with FM before treatment. Differences have been observed in various studies as regards the mean NFR threshold values in patients with FM. These differences may be explained by methodological differences in measurement or that different cultures and/or ethnicities have different pain sensitivities (21).

In a multicenter double-blind randomized trial, Crofford *et al* (6) demonstrated a significant difference in VAS values in the pregabalin group which received pregabalin at 300 mg compared with the placebo group in patients with FM. Hooten *et al* (8), in a study on 72 patients with FM, investigated the effects of aerobic and strengthening exercises on pain scores after 3 weeks, and a decrease in pain scores following treatment was reported. In the present study, the baseline VAS resting and activity scores were similar in both groups and a significant decrease was observed in both groups following treatment.

The study by Straube *et al* (22) demonstrated that pregabalin at 300, 450 and 600 mg/day was effective on the FIQ scores. Similarly, the results of the present study revealed a significant improvement in FIQ scores following treatment in both groups.

As regards signs of depression, both groups of patients exhibited a significant reduction in BDI; however, there was no significant difference between the BDI scores of the pregabalin plus exercise combination and the pregabalin only group at the 1st month following treatment. From these results, it can be inferred that exercise provides no additional benefit in the short-term, although long-term exercise therapy may become more effective as regards the BDI scores. In the study by Sañudo *et al* (23), long-term aerobic exercise was compared with combined exercise and the BDI scores were lower in the aerobic exercise group. Strengthening and stretching rather than aerobic exercises may have the effect of decreasing symptoms of depression.

It was expected, based on previous literature that describes the NFR as a physiological correlate of pain, that a reduction in pain with increasing exercise intensity would be associated with higher NFR thresholds and lower NFR responding (24). To the best of our knowledge, the present study was the first to assess the effects of exercise (treadmill, aerobic exercises, strengthening, stretching and relaxation exercises) involving the NFR of muscles. Contrary to the initial expectations, no significant differences between the groups were found in the NFR threshold. The findings demonstrated that a short-term exercise program did not affect the NFR.

There are some important limitations to the present study that should be mentioned. The main limitation of the present study was the small sample size. Therefore, acknowledging this as a preliminary study, there is a need for further studies using a long-term exercise program to determine the efficacy of exercise.

In conclusion, the present study demonstrated that both treatment groups had a significant symptomatic improvement in the VAS, BDI, FIQ and NFR threshold values at the 1st month following treatment. However, short-term aerobic and strengthening exercises did not appear to provide additional benefits, which could be considered a chronic training effect. Further studies investigating long-term efficacy are thus required.

## Figures and Tables

**Table I:** Demographic characteristics of the patients with fibromyalgia in the present study.

Parameter	Group 1 (pregabalin) (n=18)	Group 2 (pregabalin + exercise) (n=14)	P-value
Age (years)			
Mean ± SD	42.8±2.4	43.3±2.5	0.559
Median (min-max)	43 (38-48)	43 (38-48)	
Height (cm)			
Mean ± SD	158.0±3.2	158.3±3.0	0.791
Median (min-max)	158 (152-164)	158 (154-164)	
Weight (kg)			
Mean ± SD	72.1±5.4	72.8±5.6	0.708
Median (min-max)	72 (62-82)	72.5 (62-82)	
BMI (kg/m^2^)			
Mean ± SD	28.9±2.7	29.0±2.3	0.877
Median (min-max)	28.6 (24.8-34.5)	28.8 (25.8-34.5)	
Disease duration (months)			
Mean ± SD	71.3±29.3	70.2±28.6	0.920
Median (min-max)	72 (6-132)	72 (12-132)	

Data were analyzed using the Mann-Whitney U test. BMI, body mass index.

**Table II:** Group comparisons at baseline and at the 1st month after treatment.

	Group 1		Group 2		
Parameter	Mean ± SD	Median (min-max)	Mean ± SD	Median (min-max)	P-value^b^
VAS resting					
Baseline	6.0±1.3	6 (4-8)	6.0±1.4	6 (4-8)	0.889
At 1st month	4.3±1.0	4.5 (3-6)	3.5±1.0	3.5 (2-5)	0.048
P-value^c^	#x003C;0.001^a^		#x003C;0.001^a^		
VAS activity					
Baseline	7.5±1.1	8 (6-9)	7.4±1.2	7 (6-9)	0.867
At 1st month	5.8±0.9	6 (5-7)	5.0±1.1	5 (4-7)	0.022
P-value^c^	#x003C;0.001^a^		#x003C;0.001^a^		
FIQ					
Baseline	62.5±4.9	62.5 (54.4-70.7)	62.3±4.8	62.6 (53.6-71.2)	0.937
At 1st month	53.5±5.0	52.7 (46.6-63.6)	51.2±4.5	51.0 (42.3-60.2)	0.166
P-value^c^	#x003C;0.001^a^		#x003C;0.001^a^		
BDI					
Baseline	24.0±2.6	23 (21-29)	23.8±2.7	24 (20-28)	0.838
At 1st month	13.7±2.6	13 (10-19)	13.5±2.4	13.5 (10-17)	0.833
P-value^c^	#x003C;0.001^a^		#x003C;0.001^a^		
Tender points					
Baseline	14.5±1.6	14 (12-17)	14.8±1.7	15 (12-17)	0.559
At 1st month	12.0±1.5	12 (10-14)	11.8±1.6	12 (10-15)	0.726
P-value^c^	#x003C;0.001^a^		#x003C;0.001^a^		
NFR threshold					
Baseline	11.5±4.4	11.5 (2.4-18.4)	10.8±3.4	12 (4.2-16)	0.610
At 1st month	13.4±3.4	13.5 (8-20)	12.8±2.3	13.5 (10-16.2)	0.555
P-value^c^	#x003C;0.001^a^		#x003C;0.001^a^		

BDI, Beck's Depression Inventory; FIQ, fibromyalgia impact questionnaire; NFR, nociceptive flexion reflex; VAS, visual analogue scale.

^a^P#x003C;0.05, indicates significant differences.

^b^Data were analyzed using the Mann-Whitney U test;

^c^Data were analyzed using the Wilcoxon signed-rank test.

**Table III:** Correlations of NFR scores with clinical parameters obtained before and at 1 month after treatment.

Parameter	VAS resting	VAS activity	FIQ	BDI
All the patients				
Baseline	P#x003C;0.001^b^	P#x003C;0.001^b^	P#x003C;0.001^b^	P=0.05^a^
NFR scores	Rho=-0.62	Rho=-0.69	Rho=-0.60	Rho=-0.35
Group 1				
At 1 month	P#x003C;0.001^b^	P=0.007^b^	P=0.002^b^	P=0.13
NFR scores	Rho=-0.82	Rho=-0.61	Rho=-0.68	Rho=-0.37
Group 2				
At 1 month	P=0.07	P=0.01^b^	P=0.009^b^	P=0.66
NFR scores	Rho=-0.49	Rho=-0.66	Rho=-0.66	Rho=-0.51

Data were analyzed using Spearman's correlation coefficient and relevant P-values and Rho values are presented.

^a^Indicates a significant correlation (P#x003C;0.05);

^b^indicates a highly significant correlation (P#x003C;0.01). BDI, Beck's Depression Inventory; FIQ, fibromyalgia impact questionnaire; NFR, nociceptive flexion reflex; VAS, visual analogue scale.

## Data Availability

The datasets used and/or analyzed during the current study are available from the corresponding author on reasonable request.
